# *In vitro* and *in vivo* effects on neural crest stem cell differentiation by conditional activation of Runx1 short isoform and its effect on neuropathic pain behavior

**DOI:** 10.3109/03009730903572065

**Published:** 2010-03-10

**Authors:** Nadezda Kanaykina, Klas Abelson, Dale King, Anna Liakhovitskaia, Silke Schreiner, Michael Wegner, Elena N. Kozlova

**Affiliations:** ^1^Department of Neuroscience, Neuroanatomy, Uppsala University Biomedical Center, UppsalaSweden; ^2^Department of Neuroscience, Comparative Medicine, Uppsala University Biomedical Center, UppsalaSweden; ^3^Department of Experimental Medicine, University of Copenhagen, CopenhagenDenmark; ^4^Institute for Stem Cell Research, University of Edinburgh, EdinburghUK; ^5^Department of Biochemistry, University of Erlangen-Nuremberg, ErlangenGermany

**Keywords:** Development, gene regulation, neuroglia, pain, sensory neuron, transcription factor

## Abstract

**Introduction:**

Runx1, a Runt domain transcription factor, controls the differentiation of nociceptors that express the neurotrophin receptor Ret, regulates the expression of many ion channels and receptors, and controls the lamina-specific innervation pattern of nociceptive afferents in the spinal cord. Moreover, mice lacking Runx1 exhibit specific defects in thermal and neuropathic pain. We investigated whether conditional activation of Runx1 short isoform (Runx1a), which lacks a transcription activation domain, influences differentiation of neural crest stem cells (NCSCs) *in vitro* and *in vivo* during development and whether postnatal Runx1a activation affects the sensitivity to neuropathic pain.

**Methods:**

We activated ectopic expression of Runx1a in cultured NCSCs using the Tet-ON gene regulatory system during the formation of neurospheres and analyzed the proportion of neurons and glial cells originating from NCSCs. In *in vivo* experiments we applied doxycycline (DOX) to pregnant mice (days 8–11), i.e. when NCSCs actively migrate, and examined the phenotype of offsprings. We also examined whether DOX-induced activation of Runx1a in adult mice affects their sensitivity to mechanical stimulation following a constriction injury of the sciatic nerve.

**Results:**

Ectopic Runx1a expression in cultured NCSCs resulted in predominantly glial differentiation. Offsprings in which Runx1a had been activated showed retarded growth and displayed megacolon, pigment defects, and dystrophic dorsal root ganglia. In the neuropathic pain model, the threshold for mechanical sensitivity was markedly increased following activation of Runx1a.

**Conclusion:**

These data suggest that Runx1a has a specific role in NCSC development and that modulation of Runx1a activity may reduce mechanical hypersensitivity associated with neuropathic pain.

**Abbreviations**
bFGF: basic fibroblast growth factor
bTUB: beta-tubulin
DOX: doxycycline
DRG: dorsal root ganglion
EGF: epidermal growth factor
EGFP: enhanced green fluorescent protein
GFAP: glial fibrillary acidic protein
NCSC: neural crest stem cell
Ngn: neurogenin
rtTA: reverse tetracycline-regulated transactivator
TRE: tetracycline-responsive element

## Introduction

Dorsal root ganglia (DRGs) are composed of subsets of anatomically and functionally specialized sensory neurons and glial cells, which like all other neurons and glial cells of the peripheral nervous system are derived from the neural crest. The development of these cell populations is regulated by sequential expression of a limited number of transcription factors in concert with environmental components. Runt domain transcription factor signaling plays a key role in sensory neuron specification. Thus Runx3 acts to diversify an Ngn1-independent neuronal cohort by promoting the differentiation of proprioceptive sensory neurons, whereas Runx1 controls neuronal diversification within Ngn1-dependent TrkA^+^ neurons by repression of the neuropeptide Calcitonin gene-related peptide (CGRP)-expressing phenotype and induction of a Ret-expressing phenotype (1–3). The Runx1 locus generates a number of splice isoforms which may play different specific roles in cell differentiation (4). The short Runx1 isoform (Runx1a) has a strong affinity for binding to the DNA consensus sequence which is common for all types of Runx proteins but lacks a transactivation domain. Runx1a is therefore commonly viewed as a functional inhibitor of the Runx1 long isoform (Runx1b). However, Runx1a may also have specific effects of its own, as evidenced by its ability to transactivate the interleukin-3 promoter (5) and enhance engraftment capacity of hematopoietic progenitor cells (6).

Here, we have examined the role of Runx1a in differentiation of neural crest stem cells (NCSCs) *in vitro* and *in vivo* and in the development of neuropathic pain behavior in adult mice. For this purpose we have employed the Tet system (7) to conditionally activate Runx1a in Rosa26 or Sox10-expressing cells. As a source of NCSCs we used cells from the boundary cap of Rosa26-rtTA/TRE-Runx1 or Sox10-rtTA/TRE-Runx1 transgenic mice. The boundary cap contains a population of multipotent neural crest stem cells, which are able to differentiate to sensory neurons and to Schwann cells *in vitro* (8) and *in vivo* after transplantation (9,10). The activation of Runx1a was accompanied by the expression of enhanced green fluorescent protein (EGFP), which was activated through the internal ribosome entry site (IRES)-EGFP.

## Material and methods

### Generation of transgenic mice

The tetracycline-inducible Runx1a (short isoform)-EGFP mouse was generated. At the core of the bi-partite regulatable system in this mouse is a reverse tetracycline transactivator (rtTA) which after binding tetracycline/doxycycline becomes capable of interacting with the tetracycline-responsive element (TRE). This interaction results in up-regulation of a Runx1a cDNA and subsequent IRES-EGFP expression. RtTA was targeted into the ubiquitously expressed Rosa26 locus, or Sox10 locus, and Runx1a-IRES-EGFP cDNA driven by the tetracycline-responsive element (TRE) targeted upstream the hypoxanthine phosphoribosyltransferase (HPRT) locus. By breeding Rosa26-rtTA or Sox10-rtTA mice with TRE-Runx1a mice, two strains of mice were obtained: Rosa26-rtTA/EGFP-TRE-Runx1a and Sox10-rtTA/EGFP-TRE-Runx1a.

### Animals and genotyping

All procedures were approved by the Regional Ethics Committee for Research on Animals and carried out according to the guidelines of the Society for Neuroscience.

To explore the effect of ectopic Runx1a expression during development we used new-born double transgenic Sox10-rtTA/TRE-Runx1a mice, which were obtained by breeding Sox10-rtTA2^S^-M2 mice with HPRT-Runx1a-IRES-EGFP mice. Doxycycline (DOX) was given to the pregnant dam between days 8 and 11 of pregnancy. The offsprings were genotyped individually after birth, and double transgenic pups were compared with non-transgenic ones from the same litter.

For *in vitro* experiments and for *in vivo* experiments in a neuropathic pain model we used Rosa26-rtTA/HPRT-Runx1a-IRES-EGFP mice. All mice were homozygous for the Rosa26-rtTA gene and differed only in the presence of the TRE-Runx1a gene. For the neuropathic pain experiments we used three groups of double transgenic mice: 1) without injury as a control group, 2) with injury but without DOX-activated Runx1a expression as a second control group, and 3) with injury and with DOX-activated Runx1a expression. For the *in vitro* experiments we used DOX-activated and non-activated neurospheres obtained from double transgenic mice Rosa26-rtTA/TRE-Runx1a.

Sox10-rtTA2^S^-M2 mice, which contain a second generation reverse tetracycline-controlled transactivator (rtTA2^S^-M2) knocked-into the genomic *Sox10* locus, have been previously described along with protocols for their genotyping (11). Rosa26-rtTA/TRE-Runx1a male and female mice were genotyped before breeding. Each individual embryo from which we collected DRGs for stem cell cultures was also genotyped.

Primers for genotyping mice with the Runx1 gene, Sox10-rtTA (9), were designed to specific parts of the different transgenic constructs.

### Rosa26-rtTA/HPRT-Runx1a-IRES-EGFP mice

**Figure 1.**
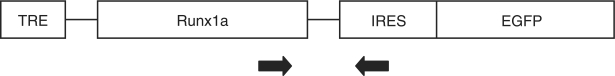
Genotyping strategy. The forward primer is located in human Runx1a, the reverse primer binds to the IRES sequence.

### Culture of boundary cap neural crest stem cells (bNCSCs)

Dorsal root ganglia (DRGs) were isolated from 11-day-old embryos (E11) and used for setting up bNCSC cultures (8). The medium was changed every other day before neurospheres began to form after about 3 weeks of culture.

### Differentiation assay

After the neurospheres were formed, they were dissociated to single cells and divided into two groups, and to one of them DOX (5 μg/mL) was added every third day during the formation of new neurospheres. Under DOX treatment EGFP-expressing neurospheres were formed. Newly formed DOX-treated EGFP-expressing neurospheres and DOX-untreated neurospheres were plated on poly-D-Lysine (PDL) (50 μg/mL) and laminin (20 mg/mL) and maintained in DMEM-F12/Neurobasal medium supplemented with N2 1:200, B27 1:100, 0.1 mM non-essential amino acids, and 2 mM sodium pyruvate. The medium was replenished every third day. DOX was continuously added to DOX-treated wells every third day, when medium was changed.

### Assessment of NCSC cultures and immunocytochemistry

The cultures were fixed at 48 hours, 1 week, and 2 weeks after DOX treatment was started. Just before cultures were fixed, we examined whether EGFP was expressed in the cultured cells in an inverted fluorescence microscope (Nikon) and photographed cells in phase contrast and in green band pass filter. Thereafter the medium was removed, cells washed with PBS and fixed in 0.15 M phosphate-buffered 4% formaldehyde (v/v), 14% saturated picric acid (v/v) for 10 minutes. Before immunocytochemical processing the fixed slides were analyzed in the fluorescent microscope again, and the complete disappearance of Tet-regulated expression of EGFP was confirmed. This was important for the possibility to use FITC and Cy2 conjugated secondary antibodies for the subsequent immunocytochemical labeling. After fixation cells were washed carefully in cold PBS for 30 minutes and pre-incubation solution added directly to the wells with the cover-slips for 1 hour. Slides were incubated with primary antibodies against bTUB (mouse monoclonal, 1:500; Covance cat#MMS-435P), GFAP (rabbit polyclonal, 1:1000; DAKO cat#Z0334), nestin (mouse monoclonal, 1:500; Immunkemi; cat#VP-N752), Sox10 (polyclonal guinea-pig, 1:1000; M. Wegner, Erlangen), Mts1/S100A4 (rabbit polyclonal, 1:700; gift from Dr Lukanidin), S100 beta (mouse monoclonal, 1:250; Sigma cat#S2532) at 8ºC overnight. After rinsing, appropriate secondary antibodies (Jackson ImmunoResearch) were applied: Cy3 conjugated donkey anti-mouse (1:500), Cy2-conjugated donkey anti-rabbit, Cy3-conjugated donkey anti-guinea-pig, and AMCA-conjugated donkey anti-rabbit (1:100). Cover-slips were mounted in 50% glycerol in PBS containing 100 mM propyl-gallate to prevent photobleaching. As negative control secondary antibodies were added to the cells without prior incubation with the primary antibodies.

For labeling of whole neurospheres, these were carefully moved directly from the well-dish to the fixative and subsequently processed for labeling as described above. FITC-conjugated donkey anti-rabbit (1:100) antibodies were used in combination with Hoechst 33342 nuclear labeling (11 ng/mL; Molecular Probes, Eugene, OR).

### Activation of Runx1a expression in development and analysis of offspring phenotype

DOX was applied in the drinking water (3 g/L with 50 g/L sucrose) to the pregnant females with Rosa26-rtTA/TRE-Runx1a or Sox10-rtTA/TRE-Runx1a background during embryonic days 8–11. The pups were examined daily after birth, and post-mortem tissue from spinal cord and DRGs removed and immersion-fixed in 0.15 M phosphate-buffered 4% formaldehyde (v/v), 14% saturated picric acid (v/v) for 4 hours. After fixation, tissue was immersed in PBS containing 15% sucrose for cryoprotection overnight. Sections were cut at 12 μm on a cryostat and labeled with antibodies to beta-tubulin (bTUB), peripherin, and glial fibrillary acidic protein (GFAP), as described above.

### Microscopic analysis of cultures

Immunolabeled material was examined in a Nikon Eclipse E800 microscope equipped with filters for separate or combined viewing of red, green, and blue fluorescence. For photography, a Nikon DXM1200F digital camera system was used. The number of cells labeled with glial fibrillary acidic protein (GFAP) and beta-tubulin (bTUB) was counted in ten optical fields after 2 weeks in cultures from six experiments from each group.

### Neuropathic pain model and behavioral assessment

#### Surgery

The surgical procedure was based on that described by Bennett and Xie (12,13). Twelve mice harboring in their genome the transgenes ROSA-rtTA and HPRT-TRE-RUNX1a-IRES-eGFP were anesthetized with isoflurane 2.5%–3% delivered in pure oxygen. Body temperature was continuously monitored and maintained at 37.5°C during the surgery. A small incision was made medially in the thigh of the right hind leg to expose the sciatic nerve. The nerve was gently separated from adjacent blood vessels and loosely constricted with three ligations, using 6-0 polyfiber suture material. Half of the 12 mice subjected to the chronic constriction injury at the sciatic nerve received doxycycline (DOX) solution in their drinking water. The other half received the injury but not the DOX treatment. In addition, a control group was included of six non-operated animals that received DOX treatment.

#### Behavioral testing

The animals' responsiveness and sensitivity to tactile stimulation was assessed 14 days after ligation. Mice were placed in a transparent plastic cage equipped with a mesh bottom, which guaranteed easy access to the paws. Prior to testing, each mouse was allowed to acclimatize to the cage for 30 min. A series of calibrated von Frey filament (Semmes Weinstein Monofilaments; Soeling, Wood Dale, IL, USA) was used to determine the 50% likelihood of a paw withdrawal response (50% threshold) using the up-and-down method of Dixon (14). Nine von Frey filaments were used with bending forces equivalent to 0.02, 0.04, 0.07, 0.16, 0.4, 0.6, 1.4, and 2.0 g. According to the Dixon paradigm, testing was initiated with the 0.4-g hair, located in the middle of the series. Each stimulus was applied perpendicularly to the plantar surface of the right hind paw with sufficient force to bend the filament for 5–6 seconds. If paw withdrawal was not observed as a response to the initial hair stimulus, a stronger (i.e. heavier) stimulus was given. On the other hand, if the initial stimulus elicited a paw withdrawal, the next weaker (i.e. weaker) stimulus was chosen. This procedure was carried out until five measurements were obtained following an initial behavioral change, or until five negative (score 0.02 g) or five positive scores (score of 2.0 g) were obtained. The resulting patterns of positive and negative responses were tabulated using the convention, X = withdrawal; 0 = no withdrawal (see Table I), and the 50% response threshold was interpolated using the formula (15): 50% g threshold = (10^[Xf+kδ]), where Xf = value (in log units) of the final von Frey hair used; k = tabular value for the pattern of positive/negative responses; and δ = mean difference (in log units) between stimuli (here, 0.25).

**Table I.** Genotyping of transgenic mice.

OLIGO name	start	len	tm	gc%	any	3'	seq
Runx1-F	694	20	59.99	55.00	7.00	3.00	AACCCTCAGCCTCAGAGTCA
IRES-R	993	20	59.99	50.00	7.00	2.00	AGGAACTGCTTCCTTCACGA

Product size: 300.

### Statistics

Statistical analyses were performed using GraphPad Prism 5.0. D'Agostino and Pearson omnibus normality test was used to determine whether data followed a Gaussian distribution. Kruskal-Wallis test was used to test whether there were any overall differences related to treatments, followed by Dunn's post-test to compare differences between groups. *P*-values less than 0.05 were considered significant.

## Results

DOX-activated ectopic expression of Runx1a induces predominantly glial differentiation in cultured NCSCs

**Figure 2.**
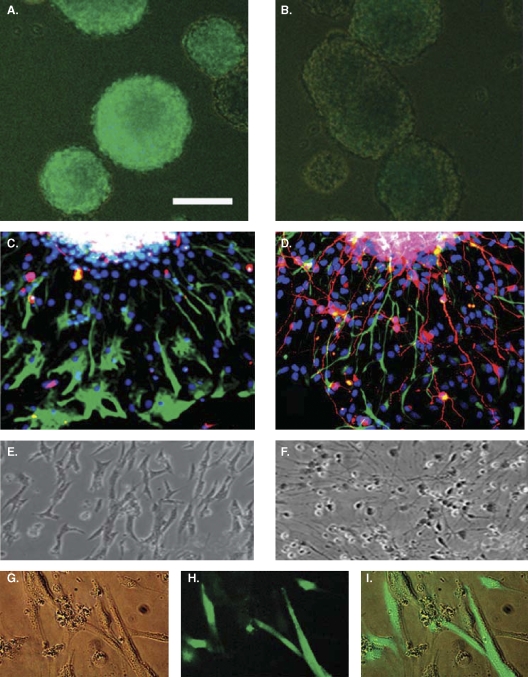
Newly formed neurospheres under doxycycline (DOX) treatment (A) and without DOX (B). Neurospheres 48 hours after removal of mitogens under DOX treatment (C) or without DOX (D). Numerous GFAP^+^ cells (green), but almost no bTUB^+^ cells (red) are present in DOX-treated neurospheres (C), whereas in untreated neurospheres numerous bTUB^+^ (red) and some GFAP^+^ (green) cells are present (D). Blue = Hoechst nuclear labeling. Culture of NCSCs after 1 week in differentiation assay. E and F: phase contrast of DOX-treated (E) and untreated (F) cultures. G–I = attached EGFP^+^ cells with glial type morphologies. Scale bar = 100 μm (A, B); 50 μm (C–I). D with permission from Lippincott Williams & Wilkins.

Under DOX treatment EGFP-expressing neurospheres were formed (Figure 2A). DOX-treated EGFP-expressing neurospheres as well as DOX-untreated EGFP-negative neurospheres (Figure 2B) were placed on differentiation assay for 48 hours, 1 week, and 2 weeks. DOX treatment was continued in previously DOX-treated cultures. After 48 hours the neurospheres had attached to the PDL/laminin-coated cover-slips and were taken for immunocytochemical analysis. bTUB and GFAP-expressing cells extensively migrated from the explants. In DOX-treated cultures most of the differentiated cells expressed the glial marker GFAP (Figure 2C), whereas in untreated cultures numerous differentiated cells expressing bTUB were found in addition to GFAP-positive ones (Figure 2D).

After 1 week in differentiation assay the morphology of DOX-treated and DOX-untreated cultures was different (Figure 2E and F). In DOX-treated cultures the majority of cells had elongated shapes resembling glial cells (Figure 2E), whereas in DOX-untreated cultures many cells resembled neurons (Figure 2F). The EGFP-labeling in live cultures was exclusively associated with elongated glia-like cells only in DOX-treated cultures (Figure 2G–I).

**Figure 3.**
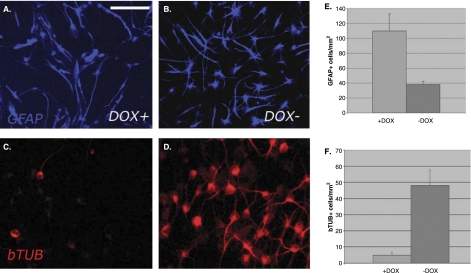
Culture of NCSCs 1 week after removal of mitogens. Doxycycline (DOX)-treated (A, C) or untreated (B, D) cultures display a striking difference in neuronal/glial relationship. In DOX-treated cultures there is an abundance of GFAP^+^ cells, and only few bTUB^+^ cells, whereas in untreated cultures GFAP^+^ and bTUB^+^ cells are both abundant. This situation is verified by the quantitative analysis of the number of GFAP^+^ and bTUB^+^ cells in DOX-treated and untreated cultures (E, F). Scale bar = 50 μm (A–D).

Two weeks after differentiation assay, cultures were fixed and labeled for GFAP and bTUB (Figure 3A–D). The proportion of GFAP or bTUB-labeled cells was counted (Figure 3E and F). The majority of DOX-treated bNCSCs differentiated to a glial phenotype (around 90% of the total number of cells), whereas untreated NCSCs differentiated to neurons and glial cells with around 50% of each type.

**Figure 4.**
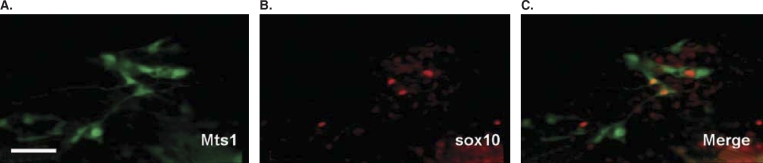
Immunocytochemistry of DOX-treated cultures, showing labeling with the glial cell markers Mts1/S100A4 (green) as well as Sox10 (red). Scale bar = 25 μm (A–C).

To further verify the identity of glial cells in the DOX-treated cultures some cover-slips were taken for immunocytochemical labeling with antibodies to Sox10 and to the calcium-binding proteins Mts1/S100A4 and S100-beta. All these glial markers were expressed in DOX-treated cultures (Figure 4).

### DOX-induced ectopic expression of Runx1a in Sox10-rtTA/TRE-Runx1a embryos results in retarded fetal growth, pigment defects, megacolon, and dystrophic DRGs

**Figure 5.**
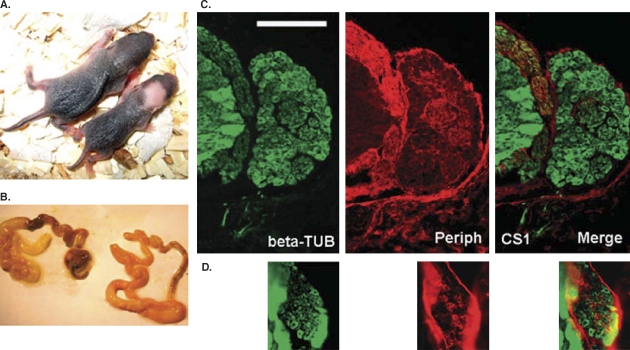
Comparison between new-born normal mice and new-born mice in which Runx1a expression was activated during embryonic days 8–11. Note the small body size and pigment defects (A, right), megacolon (B, left), and small DRG size (D compared to C) following Runx1a activation. DRGs are labeled with the neuronal markers beta-tubulin (green) and peripherin (red). Scale bar = 100 μm (C, D).

The size of the litters was reduced in DOX-activated Sox10-rtTA/TRE-Runx1a animals. The new-born pups in these litters were unusually small and displayed pigment defects (Figure 5A). Genotyping of the litters confirmed that morphological changes were manifest only in animals with dual transgenic background. These pups died after 2–3 days, presumably from gastrointestinal dysfunction, since they displayed megacolon at autopsy (Figure 5B). Microscopic analysis of immunolabeled sections from spinal cord and DRG of these pups revealed reduced sizes of DRGs (Figure 5C and D).

### DOX-induced expression of Runx1a reduces mechanical hypersensitivity in a neuropathic pain model

**Figure 6.**
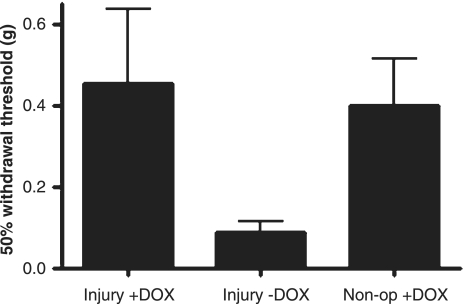
Sensitivity to von Frey filament stimulation of the hind paw assessed with the 50% withdrawal threshold in grams 14 days after a chronic constriction injury of the ipsilateral sciatic nerve.

The 50% withdrawal thresholds 14 days after surgery for each group are shown in Figure 6. All animals in the injured DOX-untreated group had a considerable increased hypersensitivity to the von Frey filament stimulation (50% threshold: 0.087 ± 0.029 g (mean ± SEM)), compared to the injured DOX-treated group (50% threshold: 0.46 ± 0.18 g) as well as to the non-operated group that received DOX (50% threshold: 0.40 ± 0.12 g).

However, due to large variations in data from the injured DOX group and the control group the differences were not statistically significant (Kruskal-Wallis statistic 5.32; *P*-value = 0.07).

## Discussion

We show that conditional activation of Runx1a in NCSCs has a powerful impact on NCSC differentiation *in vitro* and on embryonic development. Furthermore, postnatal activation of Runx1a influences the functional properties of mature injured sensory neurons.

In development of sensory ganglia Runx1 signaling is necessary first for survival of TrkA^+^ immature DRG neurons (2) and later for their diversification to non-peptidergic, Ret^+^ nociceptive, and thermoceptive sensory neurons (16). In our recent studies we observed a reduction in the numbers of non-differentiated cells in DOX-treated transplants (9), suggesting that Runx1 over-expression is sufficient to drive initial differentiation of NCSCs, but not neurogenesis per se (2). Runx1a is able to block long Runx1 isoform activity presumably via a dominant-negative mechanism, since it has a DNA binding but not transactivation capacity (17). Previously it has been shown that differentiation of glial cells from Sox10 lacZ heterozygous mice is limited (18). However, after activation of Runx1a in bNCSCs we noted predominant differentiation along the glial lineage. The reduction of neuronal differentiation could therefore be explained as a consequence of Runx1a-mediated functional inhibition of Runx1 activity. We suggest that the predominant differentiation of glial cells in our experiments may be a default mechanism at the point where neuronal and glial lineage specifications occur. The observed activation of the reporter gene EGFP specifically in glial cells suggests that Runx1 has a specific role in NCSCs differentiation. The possibility that Runx1a has effects of its own in NCSC development is supported by previous data. Runx1a was shown to transactivate the human interleukin-3 promoter, albeit with less efficiency than Runx1b (long isoform) (5). This transactivation appeared to occur at a specific consensus Runx1 binding site (TGTGGT) located on the DNAse I footprint region of the human interleukin-3 promoter and a sequence similar to the consensus binding site (TGTGGG) located in the footprint region B. Furthermore, transfection of primitive murine or human hematopoietic cells with Runx1a markedly enhanced their engraftment potential compared to cells transfected with Runx1b (6).

Thus, our findings suggest that Runx1a exerts specific effects on developing NCSCs and may support their differentiation along the glial lineage *in vitro*. The divergent properties of Runx1 isoforms, as well as their co-operation with different co-factors, therefore endow the Runx1 locus with complex regulatory effects on NCSC development and function. Whether this effect of Runx1a has a specific role during *in vivo* development and specifically in differentiation of glial cells remains to be determined.

DOX-induced activation of Runx1a in Sox10-rtTA/TRE-Runx1a embryos resulted in offspring of smaller size than normal litter-mates, and with early postnatal lethality, cutaneous pigmentation defects, megacolon, and reduced DRG size. The genotyping of pups confirmed that these defects were present only in double transgenic mice. These phenotypic characteristics suggest that ectopic transitory activation of Runx1a specifically in Sox10-expressing cells strongly interferes with normal NCSC migration and probably also their normal differentiation. These pilot *in vivo* experiments did not include the analysis of embryos at E9.5–E10 when neural crest cells are migrating. Such analysis could show if fewer cells delaminate from the neural tube in transgenic compared to wild-type mice. We can only suggest that the phenotype of the transgenic pups is a consequence of Runx1a activity, since Runx1a was induced specifically in Sox10-expressing cells during days 8–11, i.e. when NCSCs are actively migrating, and since the phenotypic changes were only observed in double transgenic Sox10-rtTA/TRE-Runx1a pups.

It was shown previously that the Sox10-rtTA heterozygous mice also display pigmentation abnormalities and megacolon although at much lower frequency and of less severity. The few Sox10-rtTA that died of megacolon usually died at the time of weaning (19), and not as early as in the present study.

Runx1 is expressed in a subpopulation of mature nociceptor neurons and has been implicated in the emergence of injury-induced neuropathic pain behavior (18). We therefore addressed the issue whether activation of Runx1a in adult mice would attenuate hypersensitivity to mechanical stimulation 2 weeks following a sciatic nerve chronic constriction injury (CCI), a well established model of neuropathic pain behavior (12,13).

We treated control (non-operated) animals with DOX to ensure that DOX per se did not have any adverse effect on the animals or on the test results. In the CCI-operated animals not treated with DOX, we observed hypersensitivity to mechanical stimulation of the hind paw. In operated animals receiving DOX, on the other hand, the withdrawal threshold was similar to that of non-operated animals. This observation suggests that activation of Runx1a with DOX inhibits the activity of Runx1 long isoform activity, which, in turn, attenuates the development of CCI-induced neuropathic pain behavior.

The mechanisms underlying neuropathic pain following peripheral nerve injury involve numerous mediators and signaling systems (20), only some of which appear to be regulated by Runx1 (18). Previously it has been shown (16) that Runx1-/- mice do not develop mechanical allodynia in a neuropathic pain model, suggesting that Runx1 function is necessary for the manifestation of neuropathic pain responses. In our experiments after activation of Runx1a short isoform, the response to mechanical stimulation was similar to that previously described in Runx1-/- mice (16). These findings suggest that modulation of Runx1 activity with Runx1a or with Runx1 siRNA deserves further investigation as a potential treatment strategy for nerve injury-induced neuropathic pain.

Since our findings in the *in vitro* experiments indicate that Runx1a has effects of its own, postnatal activation of Runx1a may not act as just a functional inhibitor of Runx1 long isoform activity. Nevertheless, our results suggest that further studies are warranted to determine whether modifying Runx1 activity confers some protection against the development of injury-induced neuropathic pain behavior and whether Runx1a affects glial differentiation during *in vivo* development.
